# Live-Attenuated Vaccines Against African Swine Fever: Strategies, Lessons, and Prospects

**DOI:** 10.3390/biology15120902

**Published:** 2026-06-09

**Authors:** Chunhao Jiang, Ruojia Huang, Rui Luo, Tao Wang, Hua-Ji Qiu, Yuan Sun

**Affiliations:** 1 State Key Laboratory of Animal Disease Control and Prevention, Harbin Veterinary Research Institute, Chinese Academy of Agricultural Sciences, Harbin 150069, China; jiangchunhao05@163.com (C.J.); huangruojia2024@163.com (R.H.); luorui20210423@163.com (R.L.); wangtao07@caas.cn (T.W.); 2 College of Animal Science and Veterinary Medicine, Heilongjiang Bayi Agricultural University, Daqing 163319, China

**Keywords:** African swine fever, live-attenuated vaccines, recombination, reversion to virulence, protective efficacy

## Abstract

African swine fever (ASF), caused by African swine fever virus (ASFV), is a devastating swine disease. To date, no commercial ASF vaccine has been authorized for global marketing except in Vietnam, and emerging genotype I/II recombinant ASFV strains pose severe new challenges to ASF control. Live-attenuated vaccines (LAVs) are widely recognized as the most promising strategy for ASF control. This review systematically summarizes three conventional development strategies for ASF LAVs, dissects the molecular mechanisms of two core bottlenecks—intergenotypic ASFV recombination and vaccine strain reversion to virulence—and elaborates rational design strategies for next-generation LAVs based on cutting-edge technologies. These strategies can fundamentally mitigate the aforementioned risks, offering promising solutions for addressing the major limitations of conventional ASF LAVs.

## Simple Summary

African swine fever (ASF) is a contagious and often lethal infectious disease of domestic pigs and wild boar caused by African swine fever virus (ASFV), posing a severe threat to the global pig industry. Although commercial ASF vaccines have been authorized for use only in Vietnam, their field deployment remains constrained by two critical limitations: emerging genotype I/II recombinant ASFV strains have completely abrogated the protective efficacy of existing genotype II-based vaccines against heterologous challenge, and all current vaccine candidates carry an inherent risk of reversion to virulence. Here, we summarize the developmental history of ASF live-attenuated vaccines (LAVs), analyze how viral evolution drives vaccine escape, and propose multiple next-generation development solutions that can effectively mitigate these risks. This review aims to support the development of safe, effective, and broad-spectrum ASF vaccines to help control the disease globally, stabilize pig production, and protect food security and rural livelihoods.

## 1. Introduction

African swine fever (ASF) is a contagious and often lethal infectious disease of domestic pigs and wild boar caused by African swine fever virus (ASFV). Since its first report in Kenya in 1921, the disease has predominantly been endemic in Sub-Saharan African countries. The first major ASF epizootic outside Africa occurred on the Iberian Peninsula, with initial outbreaks in Portugal (1957) and Spain (1960). The disease remained endemic in the region until 1995, and during this period, it spread to multiple European countries, the Caribbean, and Brazil [1]. An outbreak of ASF occurred in Georgia in 2007, and subsequently, the epidemic spread rapidly to the entire Caucasus region and Russia [2]. ASF was introduced into most Eastern European countries in 2014, showing a sustained trend of expanding epidemic spread [3]. In August 2018, ASF emerged in Shenyang, China, and spread to 21 provinces within just over three months, causing devastating losses to the Chinese pig industry [4]. In 2023, a Chinese group first reported the genotype I/II recombinant ASFV; this strain exhibits characteristics of high pathogenicity and rapid transmission, posing new and severe challenges to ASF prevention and control efforts in China [5]. Notably, this genotype I/II recombinant strain has a prolonged incubation period of 14–21 days, with approximately 30% of infected swine presenting atypical clinical signs, which further exacerbates the risk of undetected epidemic transmission [6]. Vaccination is the most effective strategy for controlling ASF, yet the unique biological characteristics of ASFV have long posed fundamental barriers to vaccine development. ASFV is a large enveloped double-stranded DNA virus and the only known DNA arbovirus. It has a complex multilayered icosahedral structure, with a 170–194-kb genome encoding more than 150 viral proteins. Most of these proteins are involved in host immune modulation, which remains the primary obstacle to ASF vaccine development [7].

Faced with the severe challenges of ASF prevention and control, the development of safe and effective vaccines has become the top priority in global ASF control. To date, live-attenuated vaccines (LAVs) represent the most advanced technical route for ASF vaccine development, with multiple candidates having completed rigorous preclinical safety and efficacy evaluations and one candidate approved for commercial use in Vietnam [8,9]. In contrast, inactivated vaccines, subunit vaccines, nucleic acid vaccines, and virus-vectored vaccines remain predominantly at the experimental or laboratory research stage, and no candidates based on these platforms have entered formal clinical trials in the regulatory sense. Despite their favorable safety profiles, these non-live vaccine platforms consistently fail to provide adequate protection in real-world field settings. Inactivated vaccines feature a well-established manufacturing process and excellent safety profiles [10]. However, inactivated vaccines fail to consistently induce detectable levels of classical virus-neutralizing antibodies against ASFV—a limitation shared by most other non-live vaccine platforms. Notably, the role of neutralizing antibodies in ASF protective immunity remains highly controversial, with compelling evidence indicating that protection against ASFV relies predominantly upon cell-mediated immunity and non-neutralizing antibody functions, such as opsonization and antibody-dependent cellular cytotoxicity (ADCC) [11,12]. Consequently, even with potent adjuvants, inactivated vaccines exhibit only incomplete and short-lived protective efficacy against ASFV challenge [13]. Crucially, only replicating LAVs can elicit the robust, sustained cellular immunity required for effective protection against ASF, a level of immunity that has not yet been reliably achieved by other non-replicating vaccine platforms [14]. Subunit vaccines differ most from inactivated vaccines in that they contain viral antigenic components but not the entire pathogen, thus offering high safety yet limited immunogenicity and weak protective capacity—the protection rate and duration of subunit vaccines fail to meet field requirements [15]. Nucleic acid vaccines feature flexible design and can induce both humoral and cellular immunity, but suffer from *in vivo* delivery inefficiency and expression instability [16]. Virus-vectored vaccines exhibit favorable immunogenicity, but potential recombination risks and limited cross-protection (defined as the ability of a vaccine to confer protective immunity against ASFV strains of different genotypes, including emerging intergenotypic recombinant strains derived from distinct parental genotypes) restrict their clinical translation [17]. In contrast, LAVs are attenuated via natural isolation, cell passage, or genetic engineering, inducing strong, long-lasting immune responses with excellent homologous protection (homologous protection is operationally defined as the ability of a vaccine to confer protective immunity against ASFV strains belonging to the same genotype, including different field isolates within the same genotype) [18]. Among the viral determinants of virulence, the multigene family (MGF) proteins, which are predominantly located in the variable genomic regions, have been identified as central regulators of host range, immune evasion, and viral pathogenesis, and thus the genes encoding these proteins represent prioritized targets for the rational attenuation of ASF vaccines [19]. Additionally, after optimizing multiple gene deletions using gene-editing technologies, such as clustered regularly interspaced short palindromic repeats and CRISPR-associated protein 9 (CRISPR-Cas9), the risk of reversion to virulence is significantly reduced [20]. Nevertheless, a comprehensive understanding of the multifaceted innate and adaptive immune responses elicited by ASFV infection remains a critical prerequisite for the rational design of next-generation LAVs [21]. Preclinical and field trials have confirmed a protection rate over 90% for LAVs, making them the most promising technical route to date [22].

This review aims to systematically summarize the research progress of the three conventional strategies for ASF LAVs. Meanwhile, it focuses on the potential risks of genetic recombination and reversion to virulence in current LAVs, attempting to decipher the underlying molecular mechanisms of these two risks. On this basis, it further proposes the development direction of next-generation ASF LAVs, aiming to provide references for the research and development of ASF vaccines.

## 2. Brief History of ASF LAVs

**Figure 1:**
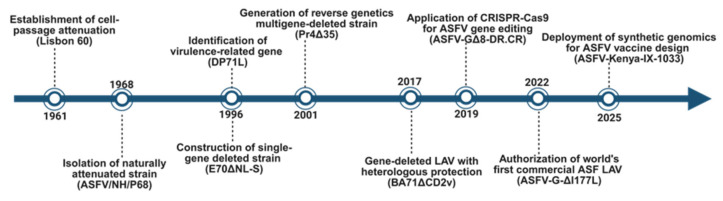
Milestones in research and development of ASF LAVs. The timeline highlights landmark discoveries and technical breakthroughs in ASF LAV development from 1961 to 2025. Abbreviations: ASF, African swine fever; LAVs, live-attenuated vaccines; ASFV, African swine fever virus; CRISPR-Cas9, clustered regularly interspaced short palindromic repeats and CRISPR-associated protein 9. This figure was created using BioRender (https://www.biorender.com/ (accessed on 30 May 2026)).

The key milestones in ASF LAV development from 1961 to 2025 are summarized in Figure 1, which chronologically presents the landmark discoveries, technical breakthroughs, and the first commercial authorization of ASF vaccines across three conventional attenuation strategies.

### 2.1. Three Conventional Strategies for the Development of ASF LAVs

Derived from wild-type strains, LAVs can replicate *in vivo* at a relatively low dose, thereby inducing a robust immune response. Based on the origin of attenuated strains, three conventional attenuation strategies have been developed for ASF LAVs, with distinct molecular mechanisms and biological characteristics. First, the natural attenuation strategy; second, the passage attenuation strategy; and third, the genetically engineered attenuation strategy, each elaborated in detail as follows.

After ASFV circulates in a certain region for many years, it may evolve into naturally attenuated strains due to genomic deletions or mutations. These strains typically do not cause fatal infection in pigs and usually induce chronic or subclinical signs. Notably, some ASFV naturally attenuated strains can confer effective protection to pigs, thus being further studied as vaccine candidates [23]. To date, OURT88/3 and NH/P68 from Portugal are the two most extensively studied naturally attenuated ASFV strains, both belonging to genotype I. OURT88/3 was isolated from soft ticks on farms, while NH/P68 was derived from domestic pigs with chronic clinical signs, and both exhibit a non-hemadsorbing phenotype [24,25]. Pigs immunized with OURT88/3 are protected against the parental strain OURT88/1, and the efficacy varies with the inoculation route—intranasal inoculation exhibits superior efficacy compared with intramuscular inoculation [26]. Although some swine immunized with NH/P68 display chronic clinical signs, they still achieve complete protection against the parental strain L60. To date, these strains have only completed small-scale inoculation trials, and whether they can provide consistent protection against heterologous virulent ASFV strains remains to be further verified in large-scale field trials [27].

In contrast to naturally attenuated LAVs formed by spontaneous genomic variation, passage-attenuated LAVs are generated via artificial *in vitro* serial passaging to induce random genomic deletions and attenuation. As early as the 1960s, there were research reports on attenuating ASFV through serial passaging in primary cells. It was found that after 70 passages of the ASFV 1455 strain in primary bone marrow cells (BMCs), the virus exhibited significant attenuation, and immunized swine could be protected against challenge with the parental virus [28]. Currently, ASFV can be passage-attenuated through BMCs, Vero cells, COS cells, MS cells, and other cell types. For instance, serial passage of a Thai ASFV isolate in MA-104 cells yielded the cell-adapted strain caASFV001-MA52, which harbors extensive *MGF* deletions in both the left and right variable regions and conferred 70–100% protection against homologous challenge in a dose-dependent manner [29]. However, the ASFV genome also undergoes changes during the passage process. Mutations accumulated by ASFV to adapt to *in vitro* cell culture conditions exhibit randomness and unpredictability, which may lead to unexpected alterations in critical immunogenic proteins and affect the quality and breadth of the immune responses. Meanwhile, impaired genomic stability also increases the risks of reversion to virulence and the genetic recombination of passage-attenuated strains during *in vivo* passaging [30,31,32,33]. Therefore, the long-term safety of this strategy should be systematically verified in animal models that simulate the complex epidemiological settings in the field.

Unlike the random attenuation of the above two strategies, genetically engineered attenuated LAVs achieve targeted attenuation by artificially knocking out virulence-related genes, and represent the most advanced and promising research direction to date. This category of vaccines is the most extensively studied and has achieved the greatest progress in current ASF vaccine research. ASFV-G-ΔI177L is a genetically engineered LAV co-developed by the Plum Island Animal Disease Center of the United States Department of Agriculture (USDA) and the National Veterinary Research Institute of Vietnam. Constructed based on the highly pathogenic genotype II ASFV isolated in Georgia in 2007, this vaccine was officially approved for national commercial application in Vietnam in July 2023 [34]. This represents a historic milestone in ASF vaccine development, as it is the first genetically engineered ASF LAV to advance from laboratory research to nationwide commercial application. However, subsequent large-scale field deployment and independent preclinical studies have revealed significant safety concerns with this strain: it can revert to virulence after 3–4 serial passages in swine but does not fully restore the lethal phenotype of the parental wild-type strain, and also causes a stillbirth rate of 43% in pregnant sows, allows vertical transmission to offspring, and induces viremia levels up to 10^6^ median hemagglutinating units (HAU_50_) at 28 days post-vaccination, indicating poor *in vivo* viral containment [33]. Despite these limitations, its commercialization has verified the basic field feasibility of the genetically engineered attenuation route and stimulated intensive research into optimizing this strategy to address safety vulnerabilities. Recent studies have shown that the pathogenicity and immunoevasion capacity of ASFV are not independently determined by individual genes, but arise from the complex interactions among multiple viral gene products, which collectively form an intricate intracellular regulatory network. For instance, the MGF300-2R protein was identified as a critical virulence-associated factor that suppresses the host innate immune response by promoting the TOLLIP-mediated selective autophagic degradation of IKK*α* and IKK*β* [35]. In a recent study, the *CD2v/A137R*-deleted ASFV strain HuBΔCD2vΔA137R exhibited favorable safety profiles in preclinical pig evaluations [36]. However, it is important to note that *CD2v* deletion generally enhances vaccine safety but frequently compromises protective efficacy against heterologous challenge [37]. Notably, this strain still exhibited detectable viremia levels of 10^3^–10^4^ DNA copies/mL at 27 days post-vaccination and 22 days post-challenge, which is suboptimal for a vaccine candidate intended for field use [36]. These findings highlight that vaccine safety is a comprehensive outcome of the interaction between the modified viral gene network and the host immune system, and multi-gene deletion strategies require careful balancing of safety and protective efficacy [38]. An understanding of this complexity has also spurred efforts to develop ASF vaccine platforms. Following the optimized strategy of multi-gene combined deletion, HLJ/18-7GD is a genetically engineered LAV developed by the Harbin Veterinary Research Institute. However, addressing insufficient cross-protection caused by ASFV’s high genomic variability and frequent recombination remains one of the fundamental challenges for ASF LAVs. In addition to the previously reported virulence-related genes, recent studies have confirmed that *D250R* is a novel, highly conserved virulence-related gene in major circulating ASFV genotypes [39]. The mRNA decapping enzyme encoded by this gene mediates viral immune evasion, and targeted deletion of *D250R* can markedly attenuate viral virulence while retaining its immunogenicity. Based on this target, researchers constructed two gene-deleted strains: SY18ΔD250R derived from the genotype II virulent strain, and JX23-02ΔD250R derived from the prevalent genotype I/II recombinant strain. *In vivo* experiments confirmed that both strains exhibited significant attenuation, with no mortality observed after immunization, providing novel candidate vaccine strains for the rational design of genetically engineered LAVs against ASF [39]. Therefore, the genetically engineered attenuation strategy focuses on designing a vaccine strain that can not only maintain genetic stability and clinical safety, but also induce broad-spectrum cross-protection against diverse circulating strains.

The basic characteristics of representative ASF LAV candidates obtained via the three strategies are compared in Table 1, which systematically evaluates their efficacy, cross-protection capacity, and safety profiles. With the continuous global spread and mutation of ASFV, recombination events among strains of different genotypes have become increasingly frequent, posing severe challenges to the protective efficacy of existing LAVs developed via the above three conventional strategies.

In summary, the three conventional attenuation strategies each present a distinct risk–benefit profile. Naturally attenuated strains retain intact viral architecture and broad antigenic repertoires but suffer from undefined genomic alterations and unpredictable cross-protection. Passage-attenuated strains offer a readily accessible manufacturing route, yet their random mutation landscapes introduce substantial genetic instability and ill-defined safety margins. Genetically engineered strains permit precise manipulation of virulence determinants and currently represent the most clinically advanced candidates. However, the reliance on defined genomic deletions creates recombination hotspots and narrows the spectrum of protection against divergent field variants. An inherent contradiction underlies this approach: the more precisely a gene is deleted for attenuation, the more likely the resulting genomic gap becomes a site for homologous recombination with wild-type strains. Such recombination events can generate chimeric viruses with enhanced immune evasion capacity, thereby severely compromising vaccine efficacy. This trade-off directly underpins the failure of conventional LAVs against emerging genotype I/II chimeric viruses and the subsequent clinical limitations arising from this failure.

### 2.2. Protective Efficacy of ASF LAVs Against Recombinant Strains

Challenge tests have been conducted on recombinant strains reported in China, Vietnam, and Russia, confirming that these recombinant strains exhibit strong pathogenicity and robust contact transmission capacity [5,46,47]. To date, only a limited number of studies on vaccines against recombinant strains have been published. Based on the recombinant ASFV-HN strain and using homologous recombination technology, two gene-deleted viruses, ASFV-HNΔMGF and ASFV-HNΔCD2vΔMGF, were generated; though the gene deletion led to attenuation, both failed to confer protection against the homologous strain ASFV-HN or the heterologous genotype II strain ASFV-GZ following immunization [48].

This significant reduction in protective efficacy has been consistently observed in the vast majority of published studies evaluating current LAVs against recombinant strains, with consistent findings in China, Vietnam, and Russia. In Vietnam, relevant research teams conducted experiments targeting locally circulating genotype I/II recombinant ASFV strains, selecting two widely deployed genotype II ASF LAVs, ASFV-G-ΔMGF and ASFV-G-ΔI177L, for immune protection evaluation [41]. The results showed that all swine immunized with the two vaccines died after challenge with an average survival time of less than 11 days; the viral transmission efficiency was consistent with that of the non-immunized swine, indicating a complete loss of protective efficacy [41]. A Russian group reported a locally emerging genotype I/II recombinant ASFV strain. Experimental infection confirmed that this isolate causes 100% mortality in swine within 7 days even at low doses. To date, there are no published data on the protective efficacy of locally developed LAVs against this recombinant strain [46]. At present, no breakthrough has been achieved in the development of vaccines against emerging recombinant strains, and traditional attenuation strategies based on a single-genotype backbone cannot effectively meet the prevention and control needs of circulating recombinant strains. Encouragingly, a very recent study has made a landmark breakthrough in this field: researchers constructed the attenuated strain JX23-02ΔD250R by knocking out the conserved virulence-associated gene *D250R*, using the prevalent genotype I/II recombinant field strain JX23-02 as the backbone. Challenge experiments confirmed that this strain achieved 100% protection against both the parental recombinant strain and virulent genotype II strains, with rapid viral clearance and extremely low viral loads in tissues after challenge [39]. This robust cross-protective efficacy against both the parental recombinant strain and virulent genotype II strains marks JX23-02ΔD250R as one of the most promising recent breakthroughs in ASF LAV development, particularly amid the rising threat of intergenotypic recombinant viruses [39]. However, a critical open question remains regarding the broader applicability of this backbone strategy: whether JX23-02ΔD250R can realistically confer consistent cross-protection against all 24 currently known ASFV genotypes, or if its efficacy is limited to the most recently circulating recombinant and genotype II variants, leaving genetically distinct strains from other genotypes unaddressed [27]. Given the extensive genetic heterogeneity and antigenic variability across ASFV genotypes, protective immunity induced by this backbone against contemporary circulating strains does not guarantee activity against divergent, less prevalent genotypes [27]. Systematic evaluation against geographically and phylogenetically diverse heterologous strains will therefore be essential to validate whether this platform can serve as a universal, long-term solution to ASF control, rather than a temporary measure tailored to the current epidemiological landscape [49]. In contrast, SY18ΔD250R, which was constructed based on the genotype II backbone, only provided 80% protection against the homologous strain, and completely failed to confer cross-protection against the recombinant strain even after booster immunization [39]. This study confirms that vaccines based on a single-genotype backbone cannot effectively overcome the cross-protection barrier against recombinant strains, and provides an effective vaccine candidate against the circulating genotype I/II recombinant strains, offering a brand-new direction for the development of broad-spectrum ASF LAVs [39].

### 2.3. Clinical Experience

**Figure 2:**
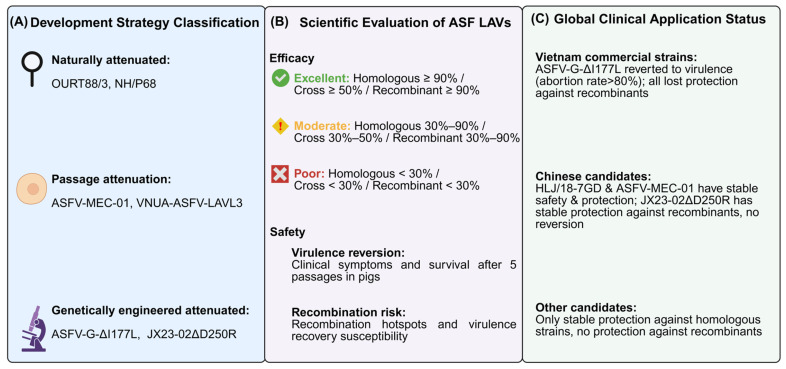
Overview of ASF LAVs. The three panels present a sequential logical framework: all vaccine candidates developed via the three conventional strategies in (**A**) must undergo systematic efficacy and safety evaluation using the standardized system in (**B**), and qualified candidates can be approved for clinical application as summarized in (**C**). Abbreviations: ASF, African swine fever; LAVs, live-attenuated vaccines; ASFV, African swine fever virus. This figure was created using BioRender.

While experimental challenge studies define the upper limit of vaccine protection under controlled conditions, late-stage clinical trials and field applications reveal practical constraints in real-world settings. First, clinical protection against emerging genotype I/II recombinant strains remains uniformly poor, even when homologous genotype II efficacy is high [41,50]. Second, clinical safety profiles differ markedly among vaccine candidates: HLJ/18-7GD and ASFV-MEC-01 exhibited favorable safety with no adverse reproductive effects or transmission in pregnant sows and after serial passage [40,44], whereas field use of ASFV-G-ΔI177L has been linked to reversion events and abortion rates exceeding 80%, despite its capacity to maintain durable antibody responses [9,42,51]. Finally, the lack of validated differentiating infected from vaccinated animals (DIVA) diagnostics continues to hinder epidemiological surveillance and disease control in vaccinated populations. The deployment of LAVs in the field necessitates a parallel DIVA strategy capable of reliably distinguishing infected from vaccinated animals, thereby enabling the accurate monitoring of virus circulation and early detection of emerging recombinant variants. Without such diagnostic tools, vaccination campaigns risk masking ongoing transmission and inadvertently facilitating the silent spread of virulent or chimeric strains. In general, current clinical evidence indicates that while ASF LAVs can confer strong homologous protection, their field utility is constrained by narrow cross-protection, strain-specific safety profiles, and diagnostic limitations. An overview of ASF LAVs is presented in Figure 2, which integrates the three conventional development strategies, the scientific evaluation system for LAVs, and the current clinical application status of major vaccine candidates.

## 3. Challenges in the Development of ASF LAVs

**Figure 3:**
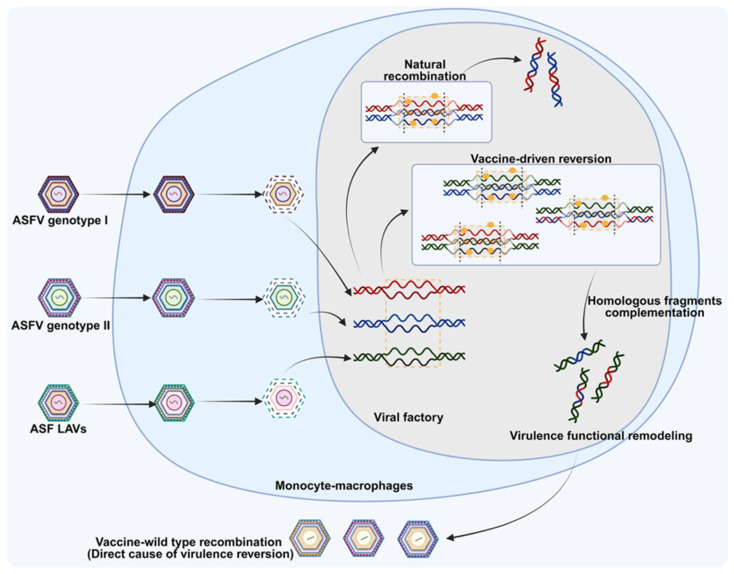
Molecular mechanisms of gene recombination and reversion to virulence in ASFV. The central biological process of homologous recombination between ASFV strains of different genotypes in monocyte-macrophages, which is the direct trigger of reversion to virulence of ASF LAVs. Black arrows indicate the sequential steps of viral infection, genome release, homologous recombination in viral factories, and progeny virus production. Yellow dots represent viral proteins associated with the recombination complex. Colored dashed boxes delineate homologous genomic fragments from different viral strains that participate in recombination events. Abbreviations: ASF, African swine fever; LAVs, live-attenuated vaccines; ASFV, African swine fever virus. This figure was created using BioRender.

The molecular mechanisms underlying the two core bottlenecks of ASF LAV development—intergenotypic recombination and vaccine strain reversion to virulence—are illustrated in Figure 3, which demonstrates that both processes are driven by homologous recombination in porcine monocyte-macrophages. The transition of ASF LAVs from experimental settings to field applications is primarily impeded by these two mechanistically linked virological phenomena. Although often discussed as separate safety concerns, both bottlenecks converge on a common molecular foundation—homologous recombination. In the context of co-infection of the same porcine macrophage, physical contact between the viral genomes of distinct genotypes or between those of vaccine and wild-type strains can initiate reciprocal fragment exchange. This process either assembles chimeric viruses with altered antigenic landscapes that circumvent vaccine-induced immunity, or restores deleted virulence determinants in attenuated backbones, thereby reconstituting pathogenicity. The following subsections dissect the mechanisms governing these recombination events and their profound implications for vaccine failure.

### 3.1. Recombination

Despite notable clinical progress in ASF LAV development and commercialization, their large-scale field application is severely restricted by two core issues arising from ASFV’s genomic plasticity: intergenotypic recombination and reversion to virulence. This section first elaborates on the molecular mechanism of ASFV intergenotypic recombination. Homologous recombination serves as the fundamental molecular mechanism for genetic exchange and the generation of chimeric recombinant strains between ASFV strains of different genotypes, and this process can occur efficiently in host cell lines [52]. *In vitro* recombination depends on co-infection within single host cells. When the multiplicity of infection (MOI) is greater than 0.1, the genomes of parental ASFV strains of different genotypes can establish physical contact within intracellular viral factories, thereby initiating homologous recombination [53]. Among these, genomic conserved regions, MGF regions and repetitive sequence regions act as recombination hotspots, with the recombination efficiency increasing in parallel with the genomic homology of parental viruses [52,54]. In current *in vitro* studies, recombination between genotypes I and II ASFV strains has garnered the most attention, which is closely linked to their status as the predominant genotypes circulating globally and their inherent co-infection potential in natural epidemic settings [5]. In contrast, the *in vitro* recombination efficiency of combinations involving genotypes VIII and X, as well as genotypes II and XVI, is notably lower, with their corresponding recombinants necessitating *in vitro* adaptive passaging to restore normal replication competence [54].

*In vivo* recombination of ASFV strains with different genotypes occurs primarily in the context of natural infections in pigs and is initiated by co-infection of its natural target cells—macrophages [55]. Its recombination efficiency is regulated not only by the genomic homology of parental viruses but also by multiple factors including host immune status, viral infection routes and *in vivo* viral load. In addition, the cellular microenvironment shaped by the viral manipulation of host processes—such as the selective autophagy pathway—may indirectly influence the likelihood of co-infection and subsequent recombination by altering viral replication fitness and immune pressure [56]. In contrast to *in vitro* recombination, the recombination hotspots *in vivo* not only encompass well-characterized loci such as genomic conserved regions, MGF regions and repetitive sequence regions, but also include additional serotype-specific loci involved in the formation of viral epitopes, namely the *EP153R* and *EP402R* gene regions [31]. It is hypothesized that this characteristic is closely associated with viral immune evasion adaptation driven by *in vivo* immune pressure. Consistent with *in vitro* research findings, recombination between genotypes I and II ASFV strains remains the most prevalent *in vivo* recombination combination. Homologous recombination of this combination proceeds efficiently under natural co-infection conditions, and the resulting chimeric recombinant strains can persist in pigs with potential transmissibility. In contrast, *in vivo* recombination involving genotypes VIII and X, as well as genotypes II and XVI, is notably infrequent [57,58]. Even when such recombination occurs, the corresponding recombinants are susceptible to clearance by the host immune system due to their inadequate adaptation to *in vivo* tissue microenvironments and host immune barriers, thereby precluding their ability to achieve efficient replication and population transmission. Furthermore, chimeric strains generated by *in vivo* recombination exhibit innate host adaptability, with their defining biological characteristics mainly manifested as follows: they have a predilection for directional replication in monocyte-macrophages of target organs; most wild-type recombinant strains display significantly enhanced virulence compared with parental strains; and the integration of antigen-associated genes leads to specific alterations in their epitopes, which can result in the reduced or even abrogated protective efficacy of existing vaccines targeting single-genotype ASFV [46].

In addition to the risk of intergenotypic recombination between wild-type strains, another core safety bottleneck restricting the field application of ASF LAVs is the reversion to virulence of vaccine strains, which is also closely related to the homologous recombination mechanism described above.

### 3.2. Reversion to Virulence

Genetic recombination between ASF LAVs and wild-type strains constitutes the central biological event triggering reversion to virulence of vaccine strains, the underlying molecular process of which is the reconstruction of pathogenicity-associated functions in vaccine strains via genomic fragment exchange [43]. The occurrence of such recombination relies on stringent intra-host co-infection conditions: residual presence or cross-border introduction of ASFV wild-type strains, such as circulating genotypes I and II strains in vaccine-immunized swine herds, enables the intracellular colocalization of vaccine and wild-type strains via the co-infection of macrophages, thereby initiating homologous recombination within viral factories [52,59]. Recombination hotspots are predominantly concentrated in the artificially modified attenuation regions, immune evasion-associated gene clusters, and epitope-encoding regions of vaccine strains, and fragment restoration or functional remodeling in these regions represents a critical prerequisite for reversion to virulence [60]. The recombination efficiency of genetically engineered attenuated strains is directly correlated with the adopted attenuation strategy: single-gene-deleted strains, with a single genomic defect, are more prone to acquiring functional fragments from wild-type strains via homologous recombination, whereas multi-gene-deleted strains, with multiple attenuation barriers, exhibit a markedly reduced risk of recombination-mediated reversion to virulence [20]. In contrast, passage-attenuated strains, characterized by random and unstable genomic variations, display a more dispersed distribution of recombination hotspots, resulting in a relatively high risk of reversion to virulence that is far more difficult to predict and control [61].

The molecular mechanisms underlying reversion to virulence can be summarized into three primary pathways: First, recombination can lead to the reconstruction of viral virulence regulatory networks. This occurs through two mechanisms: either the virulence-related genes deleted in vaccine strains are restored via homologous fragments from wild-type ASFV strains during recombination, or reversion mutations arise at key virulence loci. Such genetic changes enable the reconstruction of viral pathogenic regulatory pathways in host cells, ultimately restoring the virus to a fully virulent phenotype [33]. Second, the replication and infection efficiency of vaccine strains can be abnormally enhanced during recombination. Recombinant strains integrate tissue tropism-associated genes from co-infecting wild-type strains, which markedly improves their targeted binding capacity to monocyte-macrophages and intracellular replication efficiency. As a result, the viral load in target organs increases by 10- to 100-fold compared with the parental vaccine strains, accelerating the onset and progression of systemic pathological damage [5]. Third, recombinant strains exhibit significantly enhanced immune evasion capacity. They acquire novel epitopes from co-infecting wild-type ASFV strains, which reduces the recognition efficiency of host neutralizing antibodies and simultaneously strengthens the suppressive capacity against interferon (IFN) responses. This allows the virus to break through the vaccine-induced immune barrier and further exacerbates its pathogenicity [50]. Notably, preclinical and field data demonstrate that recombination-mediated reversion to virulence is often accompanied by a transgressive virulence phenotype. For instance, genotype I/II recombinant strains cause a 100% mortality rate in piglets, their incubation period is shortened by more than 30% compared with classical genotype II virulent strains, and they can completely evade the protection of existing genotype II LAVs, with the average survival time of immunized swine being less than 11 days post-challenge [41]. In addition, some recombination-mediated virulent revertants exhibit reproductive disorder-inducing phenotypes. For instance, virulent recombinant strains derived from ASFV-G-ΔI177L via homologous recombination can increase the abortion rate in pregnant sows to over 80%, posing dual pathogenic risks to the global swine industry [33]. Essentially, such recombination events represent the acquisition of complex evolutionary traits by the virus through genomic recombination, including enhanced pathogenicity, immune evasion and host adaptability, which pose severe challenges to the safety design of ASF LAVs and the field prevention and control of ASF [9].

Representative ASFV strains associated with natural recombination and vaccine-driven reversion to virulence are listed in Table 2, which summarizes their genetic background, core biological characteristics, and impact on existing LAVs. To address the two core challenges of intergenotypic recombination and reversion to virulence of ASF LAVs elaborated above, a series of next-generation rational design strategies for ASF LAVs based on cutting-edge technologies were developed, which can fundamentally block the molecular pathways of these two risks. These strategies are detailed in the following sections.

## 4. Rational Design of LAVs

To address the inherent limitations of conventional attenuation strategies, the development of next-generation ASF LAVs is undergoing a critical paradigm shift, moving beyond traditional gene deletion approaches toward conditional replication restriction and precision attenuation. This shift aims to fundamentally block the molecular pathways underlying intergenotypic recombination and reversion to virulence. The guiding principle is no longer simply to knock out virulence-associated genes, but rather to rewire the virus–host interactions so that viral replication is permitted only under controlled *in vitro* conditions while being stringently curtailed in the natural porcine host. These rational design frameworks aim to effectively block the molecular pathways underlying intergenotypic recombination and reversion to virulence, and have the potential to minimize these two core safety risks.

### 4.1. The Disabled Infectious Single-Cycle (DISC) Virus Platform

The design of ASF LAVs based on the DISC virus platform is focused on transforming the conventional attenuation strategy: instead of simply reducing viral virulence, it achieves conditional replication restriction. This platform uses CRISPR-Cas9 to specifically knock out viral genes that are essential for virion assembly and release in natural porcine host cells, but dispensable in Vero complementing cell lines [64]. The resulting vaccine virus can only complete its full replication cycle and achieve high-titer propagation in Vero complementing cell lines, while being unable to produce infectious progeny viruses in normal porcine host cells. Upon inoculation into swine, the virus enters its natural target, porcine alveolar macrophages (PAMs), and completes virus entry, genomic replication, transcription, and protein synthesis. This process fully mimics natural ASFV infection and thus elicits a robust immune response, and the vaccine virus is completely deprived of the capacity to produce infectious progeny virions [22]. By virtue of its replication-incompetent phenotype in natural host cells, the vaccine virus cannot produce infectious progeny, which has the potential to fundamentally block the risks of co-infection and homologous recombination with circulating wild-type ASFV. This phenotypic characteristic also effectively blocks the core molecular process of vaccine strain reversion to virulence.

Despite its theoretical advantages, the DISC virus platform remains at the proof-of-concept stage for ASF vaccine development. *In vivo* validation remains incomplete, as no DISC-based ASF vaccine candidate has been constructed and evaluated in standardized pig challenge studies to date [65]. Manufacturing feasibility poses significant challenges, as production requires stable complementing cell lines that constitutively express the deleted essential genes, which are difficult to scale up and have higher production costs compared with conventional LAVs [65]. Genetic stability remains a concern, as recombination events between the vaccine virus genome and the complementing gene in the cell line could potentially restore the full replication competence of the virus [66]. More critically, wild-type ASFV can rescue the replication-defective DISC virus through trans-complementation during co-infection: when a vaccinated swine is subsequently infected with wild-type ASFV, the wild-type virus will express the complete viral proteome, including the assembly protein deleted from the DISC virus genome [66]. This process enables the DISC virus to regenerate infectious progeny virions, which may not only spread further within pig populations, but also significantly increase the risk of recombination between vaccine and wild-type strains. Regarding regulatory requirements, global standards for replication-defective large DNA virus vaccines have not yet been harmonized. DIVA compatibility is limited, as the vaccine retains the complete viral genome except for the single deleted assembly gene and cannot be distinguished from wild-type infection by conventional serological assays [20]. For field applicability, higher immunization doses may be required to achieve sufficient immunogenicity due to the lack of viral amplification *in vivo*.

### 4.2. Targeted Protein Degradation (TPD)-Mediated Attenuation

TPD technology leverages the host ubiquitin–proteasome system, autophagy–lysosome pathway, and other cellular degradation mechanisms to specifically inactivate essential viral proteins. It breaks through the limitations of conventional gene-deletion attenuation, providing a novel and rational design strategy for tackling the fundamental challenges of reversion to virulence and genetic recombination in the development of ASF LAVs. The central principle lies in the precise insertion of proteasome-targeted degradation (PTD) tags, which can be recognized by the host ubiquitin–proteasome system, into the non-antigenic functional regions of the coding sequences of key viral proteins within the viral genome [67]. In Vero cells, selective inhibitors specifically block the function of these degradation tags to guarantee efficient propagation of the vaccine virus. Upon vaccination of the animals, the tagged essential virulence proteins of the virus are rapidly degraded by the host ubiquitin–proteasome system, which programmatically impairs viral replication competence and achieves virulence attenuation. The feasibility of this TPD-mediated vaccine design is well-supported: the MGF360 family, *I177L* and other well-validated critical proteins regulating ASFV virulence and replication are ideal targeting candidates for the TPD technology. Furthermore, the ubiquitin–proteasome system, which is widely distributed in swine, can stably recognize PTD-tagged viral proteins without the need for additional introduction of exogenous systems [68].

Notably, compared with conventional LAVs, the attenuation strategy based on TPD technology presents unique advantages in addressing the two core safety defects of current ASF vaccines. This strategy has the potential to fundamentally eliminate the risk of reversion to virulence. TPD technology does not involve large-fragment knockout of viral genomic sequences, and its attenuation effect relies on the host’s native protein degradation system rather than viral gene knockout. Therefore, even if recombination occurs between the vaccine strain and wild-type strains, the degradation regulation of virulence proteins is expected to remain intact. In parallel, TPD technology can greatly reduce the potential risk of genetic recombination. The vaccine strain constructed with TPD technology retains the complete native viral genome without artificial deletion regions, thus eliminating the recombination hotspots caused by gene knockout. The intact genome also ensures that the vaccine strain fully preserves the native antigenic profile of ASFV and induces a comprehensive immune response.

As a core antibody-mediated branch of TPD technology, the TRIM-mediated protein degradation (TRIM-Away) system has been fully validated to achieve efficient targeted degradation of ASFV proteins, providing direct experimental support for the application of TPD in ASF LAV development. Yang et al. developed a series of nanobody-based TRIM-Away constructs by fusing the RBCC domain of porcine TRIM21, a host-derived ubiquitin E3 ligase, with nanobodies that specifically recognize ASFV core structural proteins p30, p54, and p72 [69]. The study confirms that these constructs can specifically bind to target viral proteins in ASFV-infected PAMs, the natural target cells of ASFV, and degrade the target proteins through both proteasomal and lysosomal pathways. The results showed that this strategy significantly reduced the expression of viral structural proteins, decreased the viral load in infected cells by more than 100-fold, and effectively inhibited the production of infectious progeny viruses. In addition, a study by Ye et al. validated that pH240R, an ASFV protein associated with immune evasion, is also a feasible target for TPD-mediated attenuation [70], further enhancing the efficiency of host immune recognition and validating the broad applicability of this technology in ASFV control.

At present, TPD technology applied to ASF LAV development remains primarily at the *in vitro* cellular functional validation stage, and no formally published study has reported the construction of an LAV candidate verified by *in vivo* challenge experiments. Nevertheless, the conceptual feasibility of harnessing host degradation pathways for viral attenuation has been convincingly demonstrated in multiple other viral systems. For example, a PROTAR (proteolysis-targeting) influenza vaccine was generated by fusing naturally occurring C-terminal degrons to the viral M1 protein, leading to proteasome-dependent M1 degradation, robust *in vivo* attenuation, and cross-protective efficacy [71]. In parallel, an alternative TPD strategy termed LYTAR (lysosome-targeting) has been explored for influenza viruses, wherein viral proteins are conditionally degraded via the chaperone-mediated autophagy, further broadening the applicability of degradation-based attenuation [72]. These cross-viral successes highlight the significant translational promise of TPD platforms for next-generation ASF LAVs, once the unique challenges posed by the large and complex ASFV genome and its replication cycle are overcome.

However, despite these promising advances and cross-viral successes, TPD technology still faces several significant challenges that must be addressed before it can be translated into a clinically viable ASF vaccine. *In vivo* validation remains the most critical gap: the degradation efficiency of tagged proteins in the complex *in vivo* environment and the resulting immunogenicity and safety profiles are unknown. Manufacturing feasibility is challenging: identifying optimal degradation tag insertion sites that do not disrupt viral protein folding or antigenicity requires extensive screening, increasing process development time and cost. Most importantly, the insertional stability of degron tags during prolonged viral replication in the living host remains a major unresolved question. Degron tags are exogenous sequences inserted into the viral genome, and ASFV’s high replication rate and error-prone DNA polymerase increase the likelihood of deletion mutations that remove the degron tags. Such deletions would completely abrogate the attenuation effect and restore full viral virulence. To date, no studies have evaluated the long-term insertional stability of degron tags in ASFV during serial passages in swine, which is a prerequisite for clinical translation. Regulatory requirements for host degradation system-based vaccines are not yet established, as this represents a novel vaccine platform. DIVA compatibility is lacking: the intact viral genome provides no natural serological marker to distinguish vaccinated from infected animals. Field applicability may be affected by individual variations in host protein degradation system activity, leading to inconsistent immune responses across different pig populations.

### 4.3. Codon-Expansion Technology (CET) for Conditional Viral Replication

CET enables the site-specific insertion of unnatural amino acids (UAAs) into essential viral proteins via the construction of orthogonal aminoacyl-tRNA synthetase/tRNA pairs, thus achieving precise regulation of viral protein function. This codon expansion-based vaccine attenuation strategy not only preserves the native antigenic profile of the virus to induce robust and effective immune responses, but also achieves programmable inhibition of viral replication and thus virulence attenuation, thereby effectively addressing the major safety risks of reversion to virulence and homologous recombination in conventional ASF LAVs [73]. The feasibility of this technology in ASFV is further supported by comprehensive codon usage analyses: ASFV exhibits a stable, low-level codon usage bias with a clear preference for A/U-ending codons, and its genomic codon usage pattern is significantly different from that of its natural porcine host, which avoids the interference of host endogenous translation machinery on the orthogonal translation system and ensures the specificity of CET-mediated regulation [74,75].

The core mechanism of this strategy is based on the pyrrolysyl-tRNA synthetase (PylRS)/tRNA^Pyl^ orthogonal pair, a well-characterized orthogonal translation system derived from the classic methanogenic archaeon *Methanosarcina mazei* and artificially modified for high specificity. The core function of this pair is that the artificially modified PylRS specifically recognizes and loads the exogenous UAA onto its cognate tRNA^Pyl^ with no cross-reactivity with the host’s endogenous aminoacyl-tRNA synthetase/tRNA pairs, and the aminoacylated tRNA^Pyl^ enables ribosomal read-through of the amber stop codon (UAG) to synthesize full-length proteins. Systematic genomic analysis has confirmed that the ASFV genome has an extremely low frequency of endogenous UAG stop codons, and the modification of sense codons to UAG will not affect the normal translation of other viral proteins, further verifying the safety of this strategy. Briefly, specific sense codons in critical genes that are essential for ASFV replication and virulence are mutated to the UAG amber stop codon to generate the modified vaccine virus. Transgenic Vero cells stably expressing the PylRS/tRNA^Pyl^ orthogonal system are constructed in parallel, which can support the efficient propagation of the modified virus only when exogenous UAAs are supplemented in the *in vitro* culture system. In contrast, after vaccination into swine, the porcine host cells naturally lack the PylRS/tRNA^Pyl^ orthogonal system, nor are exogenous UAAs present *in vivo*. Thus, the stop codon in the viral genome triggers translation termination, the expression of full-length essential viral proteins is completely blocked, and viral replication is strictly restricted, while the expression and presentation of viral antigens are not affected. The codon usage pattern of the ASFV genome displays distinct host adaptability and high compatibility with orthogonal translation systems [70]. Transgenic Vero cells and PAMs, the natural target cells of ASFV, can satisfy the requirements for *in vitro* propagation and *in vivo* compatibility validation, demonstrating clear feasibility for application [76].

CET technology has been successfully applied to the rational attenuation of several large double-stranded DNA viruses (including pseudorabies virus, PRV), with preclinically validated technical systems, providing a solid proof-of-concept for its application in ASFV [73]. Combined with the systematic codon usage characteristics of ASFV confirmed by existing studies, CET technology has clear application potential in the development of next-generation ASF LAVs [74,77].

Compared with conventional gene-deleted LAVs, the advantage of the CET-based strategy is that it eliminates recombination hotspots caused by artificial gene knockout and has the potential to fundamentally block reversion to virulence; meanwhile, it fully retains the native antigenic profile of ASFV to ensure efficient immune induction [74]. Systematic codon usage analysis of ASFV has laid a solid theoretical foundation for the application of this technology.

However, despite its theoretical advantages and successful applications in other large DNA viruses, CET technology still faces formidable challenges that hinder its immediate translation into a commercial ASF vaccine. *In vivo* validation remains entirely lacking, as the immunogenicity, safety and genetic stability of CET-based ASF vaccines in pigs have not been evaluated. Manufacturing feasibility represents the most significant barrier, with the cost and delivery of UAAs constituting the primary bottlenecks. Commercially available UAAs currently cost $100–$10,000 per gram, which is 100 to 10,000 times more expensive than standard amino acids. For large-scale swine production, this would increase the vaccine production costs by 10- to 100-fold, making it economically unfeasible for widespread field use. Additionally, UAA delivery poses significant challenges, as most UAAs have poor cell membrane permeability and require the optimization of cell culture media formulations to improve uptake [78]. UAAs also exhibit limited stability in aqueous solutions, leading to batch-to-batch variability in vaccine yield and potency. Furthermore, the transgenic complementing cell lines required for production have lower growth rates and virus productivity compared with conventional Vero cells, further increasing manufacturing complexity and cost [79]. Genetic stability concerns include the potential for ribosomal read-through of UAG codons in the absence of UAAs or the emergence of suppressor mutations that restore full-length protein expression [80]. Regulatory requirements are extremely stringent, as UAAs are novel biological substances whose long-term biosafety in food-producing animals requires extensive evaluation, presenting major regulatory hurdles. DIVA compatibility is entirely absent, as the vaccine retains the complete viral genome with no distinguishing serological markers. Field applicability is currently severely limited by the prohibitive cost of UAAs, making large-scale field deployment economically unviable.

### 4.4. Emerging Precision Attenuation Strategies

In recent years, the in-depth interdisciplinary integration of virology with synthetic biology, structural biology, and epigenetics has spawned a series of novel attenuation strategies with unique technical advantages. Distinguished from conventional attenuation regimens and the three platform technologies described earlier, these strategies do not rely on simple gene knockout or exogenous system introduction. Instead, they originate from underlying mechanisms such as viral structure–function regulation, host epigenetic modulation, and post-transcriptional regulation of viral genes [81]. These approaches provide diversified and innovative paths for the precise design of ASF LAVs and offer new technical support for addressing core challenges, including insufficient protection against recombinant strains and the risk of reversion to virulence.

Structure-guided, AI-assisted site-directed mutagenesis targets the core challenge of balancing attenuation with immunogenicity preservation. Rational identification and targeted deletion of single virulence-associated genes—such as *MGF300-4L*—has been demonstrated to effectively attenuate ASFV while preserving robust immunogenicity, providing a foundational paradigm for precision attenuation [75]. Cryo-EM and AI-assisted prediction enable the precise identification of virulence-associated residues distinct from antigenic epitopes [82]. Whereas conventional large-fragment knockouts often disrupt protein spatial conformations and critical B/T-cell epitopes, high-resolution structural analysis allows for the precise mutation of residues governing virulence without compromising native antigenicity. Notably, the initial recognition of MGF300 family genes as critical determinants for viral replication in macrophages stemmed from observations of their recurrent deletion during cell-culture adaptation, underscoring the value of phenotypic screening in guiding targeted virulence gene discovery [83]. Recent cryo-EM structures of ASFV RNA polymerase (RNAP) in multiple conformations revealed the dynamic binding of the M1249L subunit tail within the active center, providing a precise target for attenuation [84]. Based on this structural insight, site-directed mutagenesis of specific non-epitopic residues regulating host IFN inhibition resulted in a mutant strain capable of normal replication in PAMs while exhibiting drastically reduced pathogenicity in piglets. This approach induced 100% homologous protection with no observed reversion to virulence after *in vivo* passaging. As the RNAP large subunits VRPB1 and VRPB2 are highly conserved across ASFV genotypes, this structure-guided precision method is particularly suited for rapid adaptation to emerging recombinant strains, minimizing genomic recombination hotspots and providing a foundation for broad-spectrum LAV design.

However, this strategy has notable limitations: *in vivo* validation is incomplete (~50% of ASFV proteins have unknown structures, risking off-target effects); manufacturing requires extensive validation for each mutation; genetic stability is threatened by compensatory mutations; regulatory evaluation of mutation-specific safety is strict; no DIVA compatibility; and efficacy against divergent genotypes remains unproven.

HDAC inhibitor-mediated epigenetic attenuation represents a distinct paradigm shift by applying directional selective pressure without direct genomic editing. ASFV evades host immunity by recruiting histone deacetylases (HDACs) to induce hypoacetylation of histones H3K9 and H3K14, thereby silencing type I IFN and interferon-stimulated genes [85]. Treatment with histone deacetylase inhibitors, such as sodium phenylbutyrate (NaPB), reverses this suppression, restoring the antiviral cellular microenvironment [86]. Serial passage of virulent ASFV in the presence of NaPB forces the virus to spontaneously accumulate adaptive, stabilizing mutations to survive the heightened innate immune environment. This process yields an attenuated strain that retains an intact genome and complete native antigenic profile, as it lacks artificial genomic deletions. The resulting multiple synergistic mutations confer a significantly lower risk of reversion to virulence compared with single or double gene-deleted strains [87]. This method is highly adaptable; by simply replacing the parental strain, it can be applied rapidly to different circulating genotypes, including novel recombinants, without complex gene-editing workflows.

Nevertheless, this approach has inherent flaws: *in vivo* validation is absent (no standardized pig challenge data); manufacturing relies on random mutations, leading to uncontrollable phenotypes and poor batch reproducibility; genetic stability is poor (attenuating mutations may be lost); regulatory standards for epigenetic vaccines are undefined; no DIVA markers; and field applicability is limited by imprecise attenuation control.

RNA interference (RNAi)-mediated targeted gene silencing: this post-transcriptional attenuation strategy addresses the safety concerns of recombination hotspots inherent in knockout strains. By integrating short hairpin RNA (shRNA) expression cassettes targeting essential ASFV virulence-associated genes into the viral non-coding regions, the vaccine virus constitutively produces small interfering RNAs (siRNAs). These siRNAs specifically degrade the mRNA of the targeted virulence gene in infected cells, achieving controllable attenuation while preserving the complete coding sequence of structural and immunogenic proteins [88]. Proof-of-concept studies demonstrated that siRNAs targeting the *A151R* gene reduced viral titers by up to 10^4^ and inhibited cytopathic effects by 90%. Notably, silencing *A151R* also indirectly downregulated the late structural protein p72, further impairing virion assembly. This approach allows for multi-target silencing to construct robust attenuation barriers. Since the viral genome remains largely intact without large deletions, recombination hotspots are minimized, and the complete native epitope repertoire is maintained, thereby enhancing cross-protective efficacy compared with conventional deletion mutants.

Despite these advantages, RNAi-based attenuation faces critical challenges: *in vivo* silencing efficiency varies across tissues, with no complete vaccine challenge data; manufacturing requires extensive screening of optimal target sites; genetic stability is compromised by viral target mutations and shRNA cassette deletion; regulatory concerns include off-target host toxicity; no DIVA compatibility; and field efficacy is inconsistent due to individual host differences.

**Figure 4:**
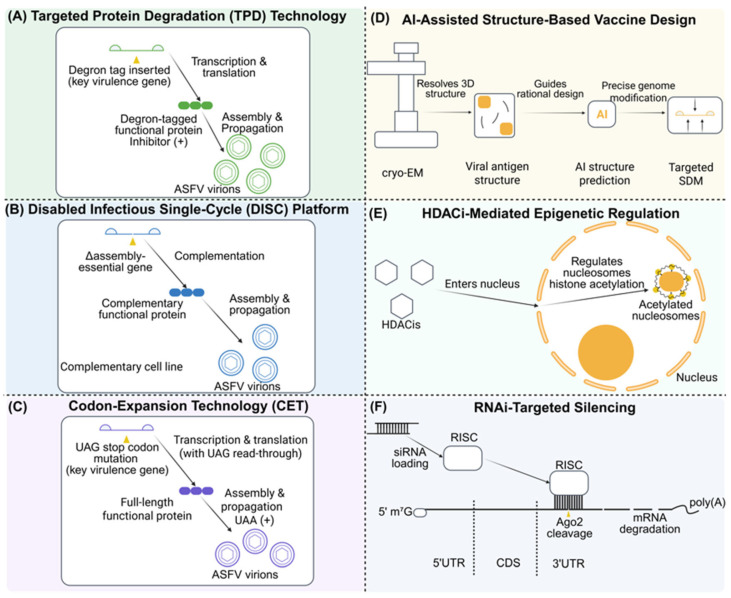
Novel design strategies for ASF LAVs. (**A**) TPD technology-based attenuation strategy. (**B**) DISC virus platform-based attenuation strategy. (**C**) CET-based attenuation strategy. (**D**) Structure-guided AI-assisted rational vaccine design. (**E**) HDACi-mediated epigenetic regulation attenuation strategy. (**F**) RNAi-targeted gene silencing attenuation strategy. Abbreviations: ASF, African swine fever; LAVs, live-attenuated vaccines; ASFV, African swine fever virus; TPD, targeted protein degradation; DISC, disabled infectious single-cycle; CET, codon-expansion technology; AI, artificial intelligence; HDACi, histone deacetylase inhibitor; RNAi, RNA interference; siRNA, small interfering RNA; RISC, RNA-induced silencing complex; Ago2, argonaute 2; UTR, untranslated region; CDS, coding sequence; SDM, site-directed mutagenesis; cryo-EM, cryo-electron microscopy; UAA, unnatural amino acid. This figure was created using BioRender.

Six emerging rational design strategies for next-generation ASF LAVs are schematically illustrated in Figure 4, which collectively represent a paradigm shift from traditional gene deletion to conditional replication restriction and precision attenuation. Recent comprehensive reviews have further highlighted the great potential of these next-generation strategies and underscored the importance of standardized evaluation systems for advancing safe and effective ASF vaccines [89].

Beyond these platform-specific limitations, the development of stable, high-titer complementing cell lines represents the most fundamental manufacturing bottleneck for both DISC and CET platforms, which depend entirely on engineered cells rather than conventional PAMs or Vero cells used for conventional LAVs. For DISC, the inherent cytotoxicity of ASFV assembly proteins precludes constitutive expression, requiring tightly regulated inducible systems that still face challenges from leaky expression [90,91]; complementing gene integration sites significantly affect expression level and stability, with random integration causing variable expression and CRISPR-Cas9-mediated targeted integration increasing development costs, while adaptation to serum-free suspension culture takes 6–12 months and often reduces virus productivity [92,93]. For CET, cell lines must stably express a complete orthogonal translation system, where balanced expression of both components is critical for efficient UAA incorporation and high virus titers, and additional optimization for UAA uptake and intracellular stability is required [78]. Finally, all complementing cell lines must undergo rigorous good manufacturing practice (GMP) validation including genetic stability over at least 20 passages, absence of adventitious agents, and consistent virus productivity, adding significant time and cost to development [94].

In general, all of the novel attenuation strategies described above offer unique complementary advantages over conventional gene knockout strategies. They can be used either alone to construct novel vaccine candidates, or in combination with existing approaches to further enhance the safety and broad-spectrum efficacy of ASF LAVs. This flexibility provides diversified technical paths for the development of next-generation ASF vaccines that can address the unmet needs of global ASF control.

Beyond these platform-specific limitations, all emerging precision attenuation strategies share several common cross-cutting challenges that must be collectively addressed to advance the field of next-generation ASF LAV development. All of the transformative approaches described above address the fundamental limitations of conventional gene-deleted LAVs, yet these approaches are still in the early phases of preclinical development, and substantial gaps persist between laboratory proof-of-concept and field-deployable vaccines. First and foremost, there is a lack of standardized and harmonized *in vivo* challenge models for evaluating vaccine efficacy and safety across different research groups. Second, all novel platforms currently suffer from high manufacturing costs and complex production processes that have not yet been optimized for large-scale commercial production. Third, the regulatory pathways for these novel vaccine platforms remain unclear and unstandardized globally, creating significant barriers to clinical translation. Fourth, there is a universal lack of integrated DIVA diagnostic systems, which are critical for effective post-vaccination surveillance and disease control. Finally, long-term field safety and efficacy data under real-world epidemiological conditions are lacking for all next-generation strategies. Addressing these collective challenges will require sustained interdisciplinary collaboration and global coordination to translate these promising technological advances into effective tools for global ASF control.

## 5. Conclusions

ASF LAVs remain a promising strategy for global ASF control, with significant progress across naturally attenuated, passage-attenuated, and genetically engineered platforms. However, two core bottlenecks persist: loss of cross-protection against emerging genotype I/II recombinant strains, and the inherent risk of reversion to virulence driven by homologous recombination between vaccine and wild-type viruses. To overcome these limitations, next-generation LAVs must prioritize three key attributes—broad-spectrum efficacy against circulating and recombinant genotypes, enhanced biological safety achieved through conditional replication restriction or precision attenuation, and compatibility with DIVA diagnostic frameworks. Emerging technologies, including DISC virus platforms, targeted protein degradation, codon-expansion-based conditional replication, and AI-guided precision mutagenesis, offer rational pathways toward these goals by preserving native antigenic architecture while eliminating recombination hotspots. Finally, sustained international collaboration in strain surveillance, standardized challenge models, and regulatory harmonization will be essential to translate these advances into field deployment and to safeguard global swine production. In summary, future development of ASF LAVs requires not only broad cross-protection against diverse genotypes, but also validated safety barriers to prevent reversion, DIVA-compatible surveillance, and rigorous field-level evaluation to ensure real-world safety and efficacy.

## Figures and Tables

**Table 1:** Basic characteristics of ASF LAV candidates obtained by different strategies.

Research Strategies	Representative Strains	Efficacy	Cross-Protection	Safety	References
Naturally attenuated	OURT88/3	100%	Genotype II: cross-protection < 30%	Only mild fever, no mortality or chronic organ damage	[26]
	NH/P68	100%	100% protection against heterologous genotype II Arm07 isolate; no cross-protection against other heterologous genotypes	Only mild fever, no mortality; mild chronic clinical signs in some swine	[24]
	Lv17/WB/Rie1	100%	92% protection against homologous genotype II Arm07 isolate; no protection against the heterologous genotype IX Ken06.Bus strain	Non-hemadsorbing; safe profile demonstrated in wild boar oral bait studies	[27]
Passage-attenuated	ASFV-MEC-01	100%	No challenge test for genotype I virulent strain	Safe for piglets and pregnant sows; no horizontal/vertical transmission; no adverse reactions after serial passaging	[40]
	VNUA-ASFV-LAVL3	100%	Not tested against heterologous strains	No clinical signs at all tested doses; cleared from blood within 14–17 days; single dose protects up to 2 months	[22]
Genetically engineered attenuated	ASFV-G-Δ*I*177L	100%	No protection against genotype I/II recombinant strains; all immunized swine died after challenge	Severe safety risks for pregnant sows; complete reversion to virulence after serial passaging in swine; abortion rate > 80% caused by virulent revertants	[33,34,41,42]
	ASFV-G-ΔMGF	100%	No protection against genotype I/II recombinant strains; all immunized swine died after challenge	No fever or organ lesions; no significant reversion to virulence after 5 passages; no obvious reproductive disorders in field application	[41,43]
	HLJ/18-7GD	100%	No protection against genotype I/II recombinant strains	Safe for pregnant sows and offspring; good genomic stability; no adverse reproductive reactions	[5,44,45]
	JX23-02ΔD250R	100%	100% cross-protection against genotype II virulent strains	Transient mild fever after immunization, no mortality; extremely low viremia and shedding, no horizontal transmission	[39]

**Table 2:** Representative ASFV strains of gene recombination and reversion to virulence.

Origins	Strains	Background	Core Characteristics	Impact on LAVs	References
Natural Recombination	ASFV-HN	Genotype I/II chimeric	100% piglet mortality at the tested challenge doses; enhanced transmissibility	No protection by genotype II LAVs	[5]
	Vietnam 2023 I/II recombinant	Local genotype I/II chimeric	100% lethal; mean survival 5.5 d; 10× higher viremia than genotype II	No protection by commercial LAVs	[41,47,62,63]
	ASFV/Primorsky 2023	Local genotype I/II chimeric	100% lethal within 7 d at low doses	/ *	[46]
Vaccine-Driven Reversion	ASFV-G-ΔI177L revertant	Vaccine strain × wild-type ASFV	Reversion after 3 to 4 passages; >80% sow abortion	No protection by parental vaccine	[33]
	ASFV-G-ΔMGF revertant	Vaccine strain × genotype I/II wild-type	Transient fever; increased shedding; no significant reversion to virulence	No protection by parental vaccine	[41,43]

* No published data available on the protective efficacy of local Russian LAVs against this recombinant strain.

## Data Availability

The analyzed data, compiled from existing literature, is publicly accessible through the respective digital object identifier. Data sharing is not applicable to this article as no new datasets were generated or analyzed.
